# Sexuality, depression and body image after breast reconstruction

**DOI:** 10.6061/clinics/2019/e883

**Published:** 2019-05-24

**Authors:** Silvania de Cassia Vieira Archangelo, Miguel Sabino, Daniela Francescato Veiga, Elvio Bueno Garcia, Lydia Masako Ferreira

**Affiliations:** 1 Departamento de Ginecologia e Obstetricia, Universidade do Vale do Sapucai, Pouso Alegre, MG, BR.; 2 Programa de Pos-graduacao em Cirurgia Translacional, Departamento de Cirurgia, Universidade Federal de Sao Paulo, Sao Paulo, SP, BR.; 3 Departamento de Ciencias Medicas, Universidade do Vale do Sapucai, Pouso Alegre, MG, BR.; 4 Disciplina de Cirurgia Plastica, Departamento de Cirurgia, Universidade Federal de Sao Paulo, Sao Paulo, SP, BR.

**Keywords:** Breast Reconstruction, Mastectomy, Sexuality, Body image, Depression

## Abstract

**OBJECTIVES:**

To evaluate the impact of breast reconstruction after mastectomy on sexual function, body image, and depression.

**METHODS:**

This cross-sectional, comparative, controlled study was conducted with 90 women between 18 and 65 years of age who had undergone either mastectomy alone (mastectomy group, n=30) or mastectomy combined with breast reconstruction (mastectomy-reconstruction group, n=30) at least one year prior to the study or who had no breast cancer (control group, n=30). Patients were assessed for sexual function, depression, and body image using the validated Brazilian-Portuguese versions of the Female Sexual Function Index, the Beck Depression Inventory, and the Body Dysmorphic Disorder Examination, respectively.

**RESULTS:**

The three groups were homogeneous for age, marital status, body mass index, and education level. The women in the mastectomy group reported significantly worse sexual function, greater depressive symptoms, and lower body image than those in the mastectomy-reconstruction and control groups. In the mastectomy group, the frequency of sexual dysfunction was significantly greater among patients without a marital partner and those with a higher level of education than among patients in the other two groups with the same characteristics.

**CONCLUSION:**

Patients who had undergone breast reconstruction after mastectomy reported better sexual function and body image and fewer depressive symptoms than patients who had undergone mastectomy alone. Sexual dysfunction was associated with the absence of a marital partner and a higher level of education and was more frequent in the mastectomy group.

## INTRODUCTION

Breast cancer is the second most common type of cancer in the general population and the most frequent cancer among women worldwide, accounting for 30% of new cancer cases each year. Breast cancer is also the most common malignant neoplasm among women in Brazil (1). It is estimated that in 2018, approximately 59,700 new cases of breast cancer will occur, corresponding to 56.33 cases per 100,000 women (1).

Breast cancer has a relatively good prognosis when diagnosed and treated promptly. However, in Brazil, breast cancer mortality rates remain high, with 12.1 deaths per 100,000 cases (1). There are still a significant number of patients with palpable breast lesions at the time of diagnosis, indicating the need to improve early diagnosis strategies, which allows physicians to determine the correct diagnosis in a timely manner, thus optimizing the chances of treatment and reducing breast cancer mortality rates (2). Despite the use of conservative techniques, mastectomy is performed in approximately 50% of cases for several reasons, including diagnosis in advanced tumour stages, tumour position, small breast size, multifocal tumour, and patient requests (3). Mastectomy is considered one of the most devastating treatments from a psychological point of view and affects self-esteem, femininity, and body image, causing more trauma than cancer itself (4).

The quality of life of patients with breast cancer is affected by factors such as pain, fear of recurrence, fatigue, depression, feelings of decreased femininity and attractiveness, and changes in body image, self-esteem, and sexuality (5-7), and these factors are especially relevant after mastectomy (8,9). Sexual quality of life, considered one of the pillars of overall quality of life, may be influenced by episodes of depression and body image disorders (10). The World Health Organization (WHO) recognizes sexual dysfunction as a public health problem and recommends research on this topic and patient treatment because of its negative impact on quality of life, its effects related to self-esteem and interpersonal relationships, and its association with frequent emotional distress (7).

Previous studies on the sexual quality of life of women diagnosed with breast cancer have found sexual dysfunction rates of up to 60% to 70% (7). Breast reconstruction has a positive impact on different aspects of quality of life, especially on body image among young women, who usually attach great importance to body image (11). Breast reconstruction may improve self-esteem without increasing the risk of relapse or delaying the diagnosis of local recurrence (7).

Thus, the aim of this study was to evaluate the impact of breast reconstruction after mastectomy on specific aspects of patient quality of life, including sexual function, body image, and depression. If the study shows that breast reconstruction after mastectomy leads to improved quality of life in the breast cancer population, the findings may contribute to the inclusion of breast reconstruction procedures in the routine treatment of patients with breast cancer in the Brazilian public health system.

## MATERIALS AND METHODS

This cross-sectional, comparative, controlled study was approved by the Research Ethics Committee of the Universidade Federal de São Paulo (UNIFESP), Brazil (approval number 2168/08), and was performed in accordance with the ethical standards of the 1975 Declaration of Helsinki and its subsequent amendments. Written informed consent was obtained from all patients prior to their inclusion in the study; patient anonymity was assured.

The study was designed to assess aspects of quality of life (*i.e*., sexual function, depression, and body image) in women who had either undergone mastectomy alone or mastectomy combined with breast reconstruction. Factors regarded as affecting the results included no evidence of active disease and the use of antidepressants. Information on breast tumour characteristics, such as tumour staging, axillary staging, treatments received, and type of surgery, is available but was not included in the study because the subgroup sample sizes were small, which did not allow for a subgroup analysis.

Sixty women who had undergone breast cancer surgery were consecutively selected and allocated to one of two groups: the mastectomy-reconstruction group (n=30) or the mastectomy group (n=30). In addition, 30 women without breast cancer who were matched for sociodemographic factors were invited to participate in the study (control group; n=30), resulting in a total sample of 90 participants in the study.

The inclusion criteria were female gender; age between 18 and 65 years; sexually active status; and previous mastectomy combined with immediate or late breast reconstruction (mastectomy-reconstruction group) or previous mastectomy alone (mastectomy group) at least one year prior to the study or no history of breast cancer (control group).

All patients were assessed for sexual function, depression, and body image using the validated Brazilian-Portuguese versions of the Female Sexual Function Index (FSFI), the Beck Depression Inventory (BDI), and the Body Dysmorphic Disorder Examination (BDDE), respectively.

As described previously (12,13), the Brazilian version of the FSFI, a measure of sexual function in women, meets all requirements for international validation and shows acceptable discriminant validity in the literature, discriminating between women with and without sexual dysfunction (12,13). The Brazilian version of the FSFI is a brief scale composed of 19 items grouped into six domains: desire, arousal, lubrication, orgasm, satisfaction, and pain. The FSFI has been successfully applied to assess sexual function in different populations (12,13).

According to the literature (14,15), the BDI measures the severity of depressive symptoms. As a self-report instrument composed of 21 items, the instrument was developed for use with psychiatric patients but has been widely used in clinical practice and research with non-psychiatric patients and in the general population. The BDI has been translated into several languages and validated in different countries. Based on BDI scores, depression can be rated as minimum (scores 0-9), light (scores 10-16), moderate (scores 17-29), or severe (scores 30-63).

The BDDE was used for the evaluation of body image. As reported in previous studies (16), the BDDE is a specific 34-item questionnaire that measures the degree of dissatisfaction with a particular part of the body, contributing to the diagnosis of body dysmorphic disorders. A total score is calculated as the sum of the ratings for all items, except items 1 to 3, 22, 33, and 34, for a maximum score of 168. A cutoff score of 66 or more indicates some degree of dissatisfaction with one's appearance.

The questionnaires were administered in the form of interviews during routine visits in the breast cancer and plastic surgery outpatient clinics of a university hospital.

### Statistical analysis

A Kruskal-Wallis analysis of variance was performed to compare age and BDDE scores among the groups. A chi-square test was carried out to compare the qualitative variables among the groups. The Mann-Whitney test was applied to compare the mean BDI scores, weight and education level between patients with or without sexual dysfunction.

The Statistical Package for the Social Sciences version 18.0 (SPSS Inc., Chicago, IL, USA) was used for data analysis. All statistical tests were performed at a significance level α of 0.05 or 5% (*p*<0.05).

## RESULTS

The groups were homogeneous for age (*p*=0.49), marital status (*p*=0.24), body mass index (BMI; *p*=0.92), and education level (*p*=0.08), as shown in Table 1.

**Table 1:** Sociodemographic characteristics of the patients.

Variables		Groups			*p*-value
		Control	Mastectomy	Reconstruction	
Mean age (years)		47.0	48.0	47.5	0.49
Education level (%)					
	<8 years	40.0	46.4	20.0	0.08
	≥8 years	60.0	53.6	80.0
Marital partner					
	Yes	76.6	66.6	56.7	0.24
	No	23.4	33.4	43.3
Chemotherapy (%)					
	Yes	_	66.5	73.3	0.77
	No	_	33.3	27.7
BMI (%)					
	18-25	29.1	30.7	40.7	0.92
	25-30	45.9	50.0	48.1
	≥30	25.0	19.3	11.2

BMI: body mass index; Reconstruction: mastectomy-reconstruction group.

The mean age of patients in the mastectomy group was 48 years (median, 49.7), and 53% of the women were between 48 and 65 years of age.

Women who had undergone mastectomy alone showed a significantly lower (*p*=0.007) total FSFI score (median, 15.4) than women who had undergone mastectomy combined with breast reconstruction (median, 21.9) and women in the control group (median, 23.6), as well as significantly lower FSFI scores in all domains, except the lubrication and satisfaction domains (Table 2).

**Table 2:** Median scores on the Female Sexual Function Index (FSFI).

Domains	Groups			*p*-value
	Control	Mastectomy	Reconstruction	
Desire	3.0	2.2	3.3	0.006*
Arousal	3.3	2.0	3.9	0.010*
Lubrification	4.0	3.0	4.5	0.090
Orgasm	4.8	2.0	4.6	0.010*
Satisfaction	4.8	4.0	4.8	0.080
Pain	5.6	3.2	3.4	0.004*
Total score	23.6	15.4	21.9	0.007*

Reconstruction: mastectomy-reconstruction group.

Kruskal-Wallis test.

* Statistical significance (*p*<0.05).

A non-significant trend (*p*=0.053) towards a higher absolute frequency in sexual dysfunction was observed in the mastectomy group (frequency, 23/30) compared to both the mastectomy-reconstruction (frequency, 15/30) and control (frequency, 15/30) groups.

Most patients in the three groups had a marital partner. However, among those without a marital partner, the frequency of sexual dysfunction was significantly higher in the mastectomy group than in the mastectomy-reconstruction and control groups (*p*=0.04).

The frequency of sexual dysfunction was also significantly greater among patients with a higher level of education in the mastectomy group (frequency, 13/15; *p*=0.03) than in the other groups.

Menopausal women aged 48 to 65 years in the mastectomy group had significantly lower FSFI scores than those in the mastectomy-reconstruction and control groups (*p*=0.002).

Patients in the mastectomy group reported significantly higher BDI scores (median, 11.1), indicating that these patients had more depressive symptoms than patients in the other two groups (*p*=0.02). A comparison among groups of patients 18-47 and 48-63 years of age showed that younger women in the mastectomy group had the highest BDI scores (*p*=0.04).

Patients in the mastectomy group also showed higher BDDE scores (lower body image) than the other subjects (*p*=0.001), although only one patient in the mastectomy group had a BDDE score of 67, which is above the cutoff score (cutoff score = 66) characterizing actual dissatisfaction with body image (Table 3).

**Table 3:** Median Beck Depression Inventory (BDI) and Body Dysmorphic Disorder Examination (BDDE) scores for the three groups.

Scales	Groups			*p*-value
	Control	Mastectomy	Reconstruction	
BDI	5. 3	11.1	3.0	0.02*
BDDE	10.0	29.5	10.0	0.01*

Reconstruction: mastectomy-reconstruction group.

Kruskal-Wallis test.

* Statistical significance (*p*<0.05).

## DISCUSSION

The diagnosis and treatment of breast cancer result in significant psychological distress, contributing to a negative perception of quality of life. Physical changes such as impaired mobility and upper limb lymphedema, chemotherapy, vasomotor symptoms, vaginal dryness, and sexual dysfunctions may also affect the quality of life of these women (8,17).

Aesthetic standards defining a woman as sensual and attractive change over time. Breasts not only play an important physiological role in some phases of a woman's life but also represent a cultural symbol of femininity, sensuality, and sexuality. When a woman undergoes mastectomy for the treatment of breast cancer, she experiences disruption of the harmony of the “perfect body”, which becomes “imperfect”, leading to several problems involving sexuality, psychological structure, self-concept, and self-image (18,19).

The increasing number of women with breast cancer may be attributed to decreasing mortality rates, increasing rates of early detection, improved treatments, and increasing incidence of new cases of breast cancer in the last decade. Approximately 61% of patients with breast cancer will survive after 5 years of diagnosis, and approximately 50% of patients worldwide will survive for at least 15 years after diagnosis (1). These women should receive support for their psychological adjustment after the surgical treatment of breast cancer (17). Previous studies have shown that the overall quality of life of patients treated for breast cancer, when free from disease, is similar to that of women without breast cancer (20) but with clinically significant differences in cognitive and functional status, fatigue, insomnia, and financial issues (21).

Relationship quality seems to be an important determinant of sexual function in women with breast cancer and can interfere with the physiological process of excitation, lubrication, orgasm, and satisfaction (8). In the present study, the frequency of sexual dysfunction among patients without a marital partner was significantly higher in the mastectomy group than in the other groups, but no significant differences in sexual dysfunction between the groups were observed among patients who had a marital relationship.

An elapsed time ≥1 year between surgery (mastectomy or breast reconstruction) and inclusion in this study was considered adequate to reduce the physical and psychological impact associated with the surgical procedure itself.

The authors acknowledge the limitation of conducting a retrospective study, but the homogeneity of the sociodemographic characteristics of the participants and the inclusion of a control group composed of healthy women contributed to making the results reliable and meaningful (22).

Breast reconstruction is an oncologically safe procedure that improves the patient's self-esteem without increasing the risk of relapse or delaying the diagnosis of local recurrence (7). The reconstruction of the breast after mastectomy may preserve a woman's self-concept and improve her body image (23) and quality of life, resulting in a less traumatic rehabilitation process compared to that for mastectomy alone (24), especially among younger women, who give greater importance to body image than older women (25).

Adequate sexual function is an important factor in the overall quality of life and life satisfaction. Considering the barriers to discussing sexual function, the use of a validated, reliable, easy-to-use scale for assessing sexual dysfunction can be helpful for evaluating outcomes and side effects of breast cancer treatments (26). The FSFI was chosen for the study because it is a tool widely used for assessing breast cancer patients in clinical and research settings. A recently published breast cancer-specific version of the FSFI (FSFI-BC) addresses topics not included in the FSFI, such as the assessment of sexual function in sexually non-active women (27). However, this version has not yet been cross-culturally validated for use in Brazil.

In this study, a trend towards higher education level was observed among patients in the mastectomy-reconstruction group, suggesting that women with higher education may be more sensitive to body image changes resulting from mastectomy, possibly due to the demands of their active social and professional lives (28). Patients who had undergone breast reconstruction reported sexual function similar to that of the controls and significantly higher than that of patients who had undergone mastectomy alone, which is consistent with previous studies (27). No significant differences in sexual dysfunction were found among younger patients in the three groups, contrasting with other research findings (27).

Patients in the mastectomy group had a median age of 49.7 years, and the majority of them were between 48 and 65 years. According to epidemiological data, menopause occurs in Brazilian women around 51 years of age (29). Considering that female life expectancy in Brazil is 72.4 years, one-third of women's lives will be spent in the menopausal transition (40-65 years of age) between reproductive life and menopause (29). The menopausal transition alone does not diminish sexual interest but affects the sexual response (excitement phase), which becomes slower and more intense because of oestrogen deprivation. The orgasmic phase becomes shorter, and pain is more frequent due to genital atrophy. Our results showed a decrease in sexual function among women 48 to 65 years of age, including the controls, which is consistent with the findings of other authors reporting on increased sexual dysfunction during the menopausal transition (30).

Patients from 18 to 47 years of age reported better sexual function than older patients, without significant differences between the groups. However, it is interesting to note that patients in the mastectomy group had a median total FSFI score <26, which is indicative of sexual dysfunction, showing how mastectomy and a possible decrease in ovarian function due to chemotherapy affect sexual function (31). Low FSFI scores in young patients who had undergone mastectomy have also been reported by other authors (31-33).

Overall, patients in the mastectomy group showed more depressive symptoms than patients in the other two groups, which was in agreement with previous studies using the BDI to assess women treated for breast cancer (6). Changes in physical appearance resulting from radical surgical procedures for the treatment of breast cancer are one of the most frequent causes of depression and low self-concept among this population, usually because patients feel less sexually attractive and less feminine (6). Depressive symptoms were more frequent among younger patients aged 18 to 47 years, showing that the combination of age and history of depression may be an important predictor of depression in this age group (32).

In this series, no significant correlation was observed between sexual dysfunction and depression, although increased depressive symptoms were observed in patients with sexual dysfunction, especially in the mastectomy group. Patients in the mastectomy group also reported worse body image than other subjects, suggesting that changes in physical appearance caused by the surgical treatment may affect sexual dysfunction. Previous studies have also reported poor body image among patients who had undergone mastectomy (34,35). In contrast, patients who had undergone breast reconstruction after mastectomy reported body image satisfaction similar to that of women in the control group, highlighting the positive impact of breast reconstruction on body image.

Although the study design did not allow an analysis of the impact of breast reconstruction on the quality of life of patients, it permitted the assessment of sexual function and associated factors, such as body image and depression, in a group of patients who had undergone breast reconstruction, and the results of the study showed the importance of personal characteristics, such as marital status, education level, and age.

The assessment of sexual function in women who lost their breast as a result of cancer treatment is an important and integral part of patient follow-up. Sexual function assessment should also be included in the evaluation of the effectiveness of different treatments for breast cancer.

Breast cancer is considered a chronic disease, with increasing incidence in recent decades. Improved diagnostic methods and less aggressive surgical treatments increase survival time and enhance the quality of survival. It is necessary and important to evaluate the patient's quality of survival and quality of life, focusing on factors such as sexuality, body image, and depression. The assessment of these factors and their impact on patient quality of life allows the implementation of support measures by a multidisciplinary team, the identification of at-risk patients, and tailored treatment based on the assessment results.

A cross-sectional design provides the opportunity to assess patients with different treatment times whose evaluation would be more difficult in a prospective study. However, we acknowledge that a prospective design would be more appropriate to evaluate the impact of mastectomy combined or not combined with breast reconstruction on the quality of life of patients, and this may be considered a limitation of the study. Small subgroup sample sizes in terms of tumour characteristics were also a limitation that prevented some subgroup analyses. Further studies that include more patients, utilize a prospective design, and involve multiple centres are necessary to extend our results.

## CONCLUSION

Patients who had undergone breast reconstruction after mastectomy reported better sexual function, better body image, and fewer depressive symptoms than patients who had undergone mastectomy alone. The results also showed that sexual dysfunction was associated with the absence of a marital partner and a higher level of education and was more frequent in the mastectomy group.
